# Intracellular fibril formation, calcification, and enrichment of chaperones, cytoskeletal, and intermediate filament proteins in the adult hippocampus CA1 following neonatal exposure to the nonprotein amino acid BMAA

**DOI:** 10.1007/s00204-014-1262-2

**Published:** 2014-05-06

**Authors:** Oskar Karlsson, Anna-Lena Berg, Jörg Hanrieder, Gunnel Arnerup, Anna-Karin Lindström, Eva B. Brittebo

**Affiliations:** 1 Department of Pharmaceutical Biosciences, Uppsala University, Box 591, 751 24 Uppsala, Sweden; 2 Department of Environmental Toxicology, Uppsala University, Norbyvägen 18A, 752 36 Uppsala, Sweden; 3 Safety Assessment, AstraZeneca R&D Södertälje, 151 85 Södertälje, Sweden; 4 National Center for Imaging Mass Spectrometry, Gothenburg, Sweden; 5 Department of Chemical and Biological Engineering, Chalmers University of Technology, Kemivägen 10, 412 96 Gothenburg, Sweden; 6 Present Address: Medical Products Agency, Box 26, 751 03 Uppsala, Sweden

**Keywords:** ALS/PDC, Alexander disease, α-Synuclein, Proteomics, TDP-43, Ubiquitin

## Abstract

The environmental neurotoxin β-*N*-methylamino-l-alanine (BMAA) has been implicated in the etiology of neurodegenerative disease, and recent studies indicate that BMAA can be misincorporated into proteins. BMAA is a developmental neurotoxicant that can induce long-term learning and memory deficits, as well as regionally restricted neuronal degeneration and mineralization in the hippocampal CA1. The aim of the study was to characterize long-term changes (2 weeks to 6 months) further in the brain of adult rats treated neonatally (postnatal days 9–10) with BMAA (460 mg/kg) using immunohistochemistry (IHC), transmission electron microscopy, and laser capture microdissection followed by LC-MS/MS for proteomic analysis. The histological examination demonstrated progressive neurodegenerative changes, astrogliosis, microglial activation, and calcification in the hippocampal CA1 3–6 months after exposure. The IHC showed an increased staining for α-synuclein and ubiquitin in the area. The ultrastructural examination revealed intracellular deposition of abundant bundles of closely packed parallel fibrils in neurons, axons, and astrocytes of the CA1. Proteomic analysis of the affected site demonstrated an enrichment of chaperones (e.g., clusterin, GRP-78), cytoskeletal and intermediate filament proteins, and proteins involved in the antioxidant defense system. Several of the most enriched proteins (plectin, glial fibrillar acidic protein, vimentin, Hsp 27, and ubiquitin) are known to form complex astrocytic inclusions, so-called Rosenthal fibers, in the neurodegenerative disorder Alexander disease. In addition, TDP-43 and the negative regulator of autophagy, GLIPR-2, were exclusively detected. The present study demonstrates that neonatal exposure to BMAA may offer a novel model for the study of hippocampal fibril formation in vivo.

**Electronic supplementary material:**

The online version of this article (doi:10.1007/s00204-014-1262-2) contains supplementary material, which is available to authorized users.

## Introduction

Protein misfolding, intracellular fibrils, and extracellular deposits of plaques are hallmarks of many neurodegenerative diseases, including Alzheimer’s and Parkinson’s disease. The nonprotein amino acid and environmental neurotoxin β-*N*-methylamino-l-alanine (BMAA) have been implicated in the etiology of neurodegenerative disease (Banack and Cox 2003; Pablo et al. 2009; Spencer et al. 1987), and behavioral deficits have been demonstrated in rodents, monkeys, and insects exposed to BMAA (Karlsson et al. 2009c; Okle et al. 2013; Spencer et al. 1987; Zhou et al. 2009). A recent in vitro study has suggested that BMAA can be misincorporated into proteins, which may result in the formation of protein aggregates in cultured cells (Dunlop et al. 2013). Using whole-body autoradiographic imaging, we have observed incorporation of radiolabelled BMAA into tissues with high protein synthesis following iv injection in mice, indicating a potential in vivo incorporation of BMAA as a false amino acid into proteins (Karlsson et al. 2009a). The access of BMAA to the adult rodent brain is, however, reported to be limited (Karlsson et al. 2009a; Smith et al. 1992; Xie et al. 2013). In contrast, autoradiographic imaging revealed that radiolabelled BMAA is transferred across the blood–brain barrier in neonatal mice, with a distinct localization in specific brain regions such as the hippocampus (Karlsson et al. 2009b).

Radiolabelled BMAA is efficiently transferred to mother’s milk in rodents, and the subsequent transfer to the suckling pup results in a high exposure to the neonatal brain (Andersson et al. 2013). BMAA is a developmental neurotoxicant that can induce a suite of changes (Engskog et al. 2013; Karlsson et al. 2009a, b, 2013a, b), including long-term learning and memory deficits (Karlsson et al. 2009c, 2011) as well as regionally restricted neuronal degeneration, necrosis, mineralization, and astrogliosis in the adult rat hippocampus (Karlsson et al. 2012). To understand the mechanisms of BMAA-induced long-term neurobehavioral alterations, more studies on the effects of BMAA in the hippocampus are needed, as this brain area is essential for learning and memory.

The aim of the present study was to further characterize long-term changes in the hippocampus of adult rats treated neonatally (postnatal days; PND 9–10) with BMAA using histopathology, IHC, transmission electron microscopy, and proteomics. The results demonstrated that BMAA elicited extensive intracellular fibril formation, neurodegeneration, astrogliosis, microglial activation, and mineralization. The BMAA-induced changes in the hippocampus were progressive, affecting more animals and displaying increased severity at later time points. Laser capture microdissection and subsequent proteomic analysis of the histopathologically altered CA1 area demonstrated that several chaperones, cytoskeletal, and intermediate filament proteins were enriched.

## Materials and methods

### Chemicals

Unless otherwise stated, all chemicals were obtained from Sigma-Aldrich Co. (St. Louis, MO). β-*N*-methylamino-l-alanine (L-BMAA) hydrochloride (≥97 %) was used.

### Experimental design

The experimental design and doses were similar to those previously reported to induce cognitive and neurodegenerative changes in adult animals (Karlsson et al. 2009c, 2011, 2012). Pregnant outbred Wistar rats were obtained from Taconic (Ejby, Denmark). Each dam was housed alone in a Macrolon cage (59 × 38 × 20 cm) containing wood-chip bedding and nesting material. The animals were maintained on standard pellet food (R36 Labfor; Lantmännen, Kimstad, Sweden) and water ad libitum and were housed in a temperature-controlled and humidity-controlled environment on a 12-h light/dark cycle. On the day of birth (PND 0), all litters were arranged to contain eight pups, with a homogeneous distribution of males and females. The male pups in each litter were randomly assigned to the control group or to one of the BMAA treatment groups. The male pups were administered one daily sc injection (20 μl/g) of BMAA 460 mg/kg (corresponding to 600 mg/kg BMAA HCl; *n* = 23) freshly dissolved in Hanks’ balanced salt solution or vehicle (*n* = 18) for 2 days on PND 9–10. After weaning on PND 22 and onward, 3 male rats were housed together in Macrolon cages in their respective treatment groups. All animal experiments were approved by the Uppsala Animal Ethics Committee and followed the guidelines of Swedish legislation on animal experimentation (Animal Welfare Act SFS1998:56) and European Union legislation (Convention ETS123 and Directive 86/609/EEC).

### Histopathology and immunohistochemistry

The animals were killed by decapitation at 2 weeks (vehicle *n* = 6, 460 mg/kg *n* = 7), 3 (vehicle *n* = 6, 460 mg/kg *n* = 8), or 6 (vehicle *n* = 6, 460 mg/kg *n* = 8) months of age. The brain samples were immersed in cold 4 % phosphate-buffered formalin (pH 7.4). After fixation, the samples were embedded in paraffin and cut in 4-µm transverse sections at a number of levels, including the olfactory bulb, frontal cortex, basal ganglia, striatum, thalamus, hippocampus, and mesencephalon, including substantia nigra and pons. Samples of the liver and kidneys of all animals were likewise processed. The brain, liver, and kidney sections were stained with hematoxylin and eosin (H&E) and examined by light microscopy. Selected brain sections were stained with Congo Red, for identification of amyloids, periodic acid-Schiff (PAS), for detection of glycogen and glycoproteins, and von Kossa’s stain and Alizarin red, for detection of calcium. The histopathological changes were scored using the following grading system: 0 = none, 1 = minimal, 2 = slight, 3 = moderate, and 4 = marked lesion.

Serial paraffin sections from the same brain levels as examined by H&E staining were used for IHC with markers for α-synuclein, glial fibrillar acidic protein (GFAP), isolectin B4, transactive response DNA-binding protein 43 (TDP-43), tau protein, tubulin, and ubiquitin. IHC for these proteins (except isolectin B4) was performed using the staining module Discovery XT. Ventana^®^ Medical Systems Inc., Tucson, AZ, USA, supplied all solutions for pretreatment, antibody dilution, detection, counterstaining, and rinsing steps. IHC for detection of isolectin B4 was performed using the staining module IntelliPATH FLX. Biocare Medical, Concord, CA, USA, supplied all solutions for antibody dilution, detection, counterstaining, and rinsing steps. Details on primary and secondary antibodies and detection systems used are provided in the Supplemental Table 1. Slides stained without primary antibody (only antibody diluent in the primary antibody step) served as negative controls. Relevant positive controls were run in parallel.

### Ultrastructural examination of neurons and astrocytes in the hippocampal CA1

Selected formalin-fixed brain samples of representative animals from both vehicle controls and BMAA-treated animals (3- and 6-month survival time points) were used for electron microscopy. The specimens were postfixed in 3 % glutaraldehyde in 0.1 M Sorensen’s phosphate buffer, rinsed in buffer, and further fixed in 2 % OsO_4_ in 0.1 M Sorensen’s buffer. After a second rinse with buffer, the sections were dehydrated in increasing concentrations of ethanol and propylene oxide and embedded in epoxy resin. The embedded specimens were trimmed, and semithin and ultrathin sections were made using a Leica UC6 ultra microtome. The semithin sections (1 μm) were stained with 0.05 % toluidine blue. The ultrathin sections (70–90 nm) were contrasted with 4 % uranyl acetate and lead citrate (Reynolds). Electron microscopy examinations were made using a Philips transmission electron microscope CM10. Images were taken with a Megaview II CCD camera and captured using the AnalySIS computer program (Soft Imaging System, Münster, Germany).

### Laser capture microdissection of the hippocampal CA1

**Fig. 1:**
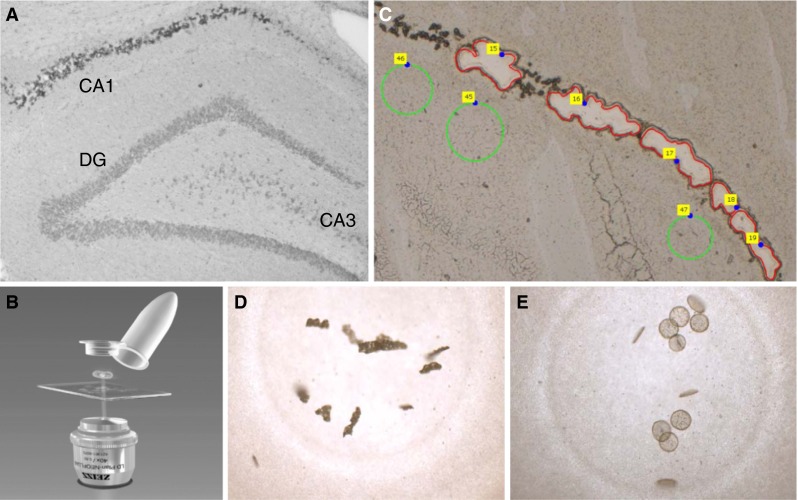
Hippocampal CA1 areas of a 6-month rat treated neonatally with BMAA on PND 9–10, showing deposition of birefringent material within neurons (**a**) were collected using laser capture dissection (**b**). Examples of collected sample areas are shown in red and control tissue in *green* (**c**). The CA1 sample (**d**) and control tissue (**e**) were catapulted into the cap of the tube and directly treated with trypsin for a two-step digestion in the capture tubes, and LC-MS/MS was performed

One additional neonatal animal was treated with BMAA (460 mg/kg) as above and killed by decapitation at 6 months of age. The distinct histopathological changes in the brain of this rat have previously been reported and are identical to the changes in BMAA-treated rats observed in the present study (Karlsson et al. 2012). Freshly prepared coronal 20-μm cryosections of the brain from the BMAA-treated animal were mounted on PEN membrane slides (Carl Zeiss Microscopy GmbH). Histopathologically altered areas of hippocampal CA1, containing birefringent material, and ultrastructurally packed with fibrils were dissected using a PALM MicroBeam AxioObserver inverted microscope (Carl Zeiss) equipped with a 355-nm pulsed laser for dissection (Fig. 1a–c) and contamination-free catapulting into the cap of two tubes (Fig. 1d, e). Adjacent histologically normal hippocampal CA1 areas were captured as control tissues (Fig. 1c). In total, 230,000 µm^2^ was collected from the sample and control area each.

### Identification of proteins enriched in the histopathologically altered hippocampal CA1 areas by proteomic analysis

The catapulted samples of the histologically altered hippocampus CA1 and adjacent histologically normal areas were directly treated with trypsin for a two-step digestion in the capture tubes (500 µL of opaque adhesive), and LC-MS/MS was performed. In brief, 25 µL of trypsin solution (0.5 µg/µL Promega sequencing grade trypsin, 200 mM triethylammonium bicarbonate, 1 % (w/v) sodium deoxycholate, 10 mM dithiothreitol) was added, and the samples were incubated overnight at 37 °C. The supernatant in the caps was removed to a maximum recovery vial, and another 25 µL of trypsin solution was added for a second round of digestion (4 h, 37 °C). Digests were combined for each sample, and the deoxycholate content was removed by acid precipitation (adding 10 µL 10 % TFA followed by centrifugation at 13,000 rpm for 10 min). Finally, the samples were desalted using Pierce C18 spin columns (Thermo Fisher Scientific, Rockford, Il, USA) and reconstituted to a volume of 15 µL in 3 % acetonitrile and 0.1 % formic acid. For sample analysis, a volume of 3 µL (i.e., 20 % of the total sample) was injected for the nanoLC–MS/MS analysis.

NanoLC-MS/MS analysis was performed using a Q Exactive–Easy-nLC 1,000 instrument combination and a set of in-house packed columns. Briefly, the analytical column was a 75-µm ID × 200 mm PicoFrit column (New Objective Inc.) packed with ReproSil-Pur C18-AQ, 3-µm particles (Dr Maisch GmbH), and the trap column was 100 µm ID × 45 mm of the same material.

Gradients of 125 min were run at a flow rate of 150 nL/min (A-solvent: 0.2 % formic acid in water and B: 100 % acetonitrile). Mass spectrometry data acquisition parameters were set according to the “sensitive” parameters proposed by Kelstrup et al. (2012), fragmenting the 12 most abundant precursors in each scan.

Protein identification was performed by searching the raw data against the UniProt Knowledgebase database (UniProtKB/TrEMBL, download date November 29, 2012) restricted by taxonomy to *Rattus* (a total of 42,567 sequences) using the 2.3.2 release of the Mascot search engine. Search parameters were in brief as follows: precursor tolerance: 10 ppm; fragment tolerance: 80 mmu; and dynamic modifications: oxidation (M), deamidation (NQ), methylthio (C), and missed cleavages: 1. Identification results were limited to peptide assignments of ≥99 % confidence as assigned by Mascot. The false-discovery rate levels (1 % FDR) were 0.0063 and 0.0083 for sample and control searches, respectively, but 0.01 was chosen as the cutoff for the comparison. Comparison between hippocampal CA1 areas with intraneuronal birefringent material (sample) and adjacent CA1 areas without birefringent material (control) was based on the number of peptides identified for an assigned protein, which is indicative of protein abundance. Proteins that were identified by more peptides in the control tissues compared with the sample as well as proteins only detected by one peptide were discarded.

To verify the presence of ubiquitin, the laser-captured material was studied directly with MALDI TOF MS. Here, the collected tissue material was resuspended in 5 µL of acetonitrile/water/TFA (50:50:0.1, v:v:v). The sample (1 µL) was spotted directly onto a MALDI target plate (MTP ground steel, Bruker Daltonics, Bremen, Germany) followed by the addition of 1 µL of matrix solution (HCCA, 30 mg/mL, 50 % ACN, 0.1 % TFA). The sample was allowed to dry and subsequently analyzed with an Ultraflextreme MALDI TOF/TOF instrument (Bruker) running in linear positive mode. The delay time was set to 250 ns, and the extraction voltage to 25 kV (IS1). The spectra were calibrated externally with calibrant peaks (Protein Standard 1, Bruker) spotted adjacent to the sample.

## Results

### Histopathology and immunohistochemistry

The vehicle-treated control animals did not display any histopathological abnormalities in the brain, liver, or kidney at any survival time.

### Two-week survival time

The BMAA-treated rats did not display any histopathological changes in the studied brain regions (Table 1), liver, or kidneys compared to the vehicle-treated control animals.

**Table 1:** Histopathological changes in the hippocampus CA1 of rats treated neonatally with BMAA on PND 9–10

Time point	Animal no.	Scoring of histopathological lesions
2 Weeks	7–13	No histopathological changes
3 Months	17, 18, 19, 23	No histopathological changes
	24	Neuronal degeneration/necrosis/mineralization: grade 3 Astrogliosis: grade 2
	25	Neuronal degeneration/necrosis/mineralization: grade 4 Astrogliosis: grade 3
	26	Neuronal degeneration/necrosis/mineralization: grade 3 Astrogliosis: grade 3
	27	Neuronal degeneration/necrosis/mineralization: grade 1 Astrogliosis: grade 1
6 Months	33, 39, 40	No histopathological changes
	31	Neuronal degeneration/necrosis/mineralization: grade 4 Astrogliosis: grade 4
	32	Neuronal degeneration/necrosis/mineralization: grade 3 Astrogliosis: grade 3
	37, 38	Neuronal degeneration/necrosis/mineralization: grade 2 Astrogliosis: grade 2
	41	Neuronal degeneration/necrosis/mineralization: grade 1 Astrogliosis: grade 1

Neonatal rats on PND 9–10 were injected sc with BMAA (460 mg/kg) and killed at various survival time points. Sections of paraffin-embedded brains were stained and used for histopathological examination. Scoring system: 0 = none, 1 = minimal, 2 = slight, 3 = moderate, and 4 = marked lesion

#### Three-month survival time

**Fig. 2:**
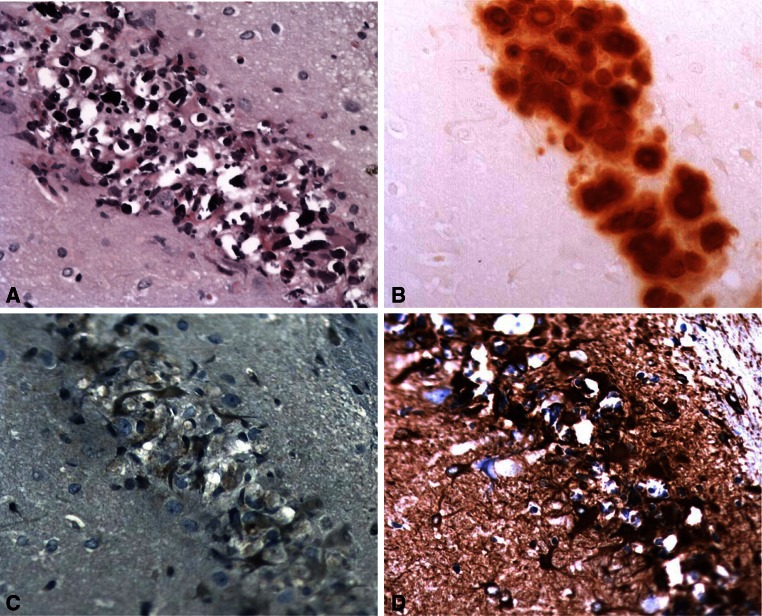
Histopathological changes in the hippocampal CA1 of rats treated neonatally with BMAA on PND 9–10, as examined at the 3-month survival time point. Degenerating/necrotic neurons contain a basophilic, birefringent material (**a**) and demonstrated to contain calcium using Alizarin *red* staining (**b**). Hypertrophic astrocytes within the same area display positive staining for ubiquitin (**c**) and GFAP (**d**). Magnification ×20

Four out of 8 rats treated with BMAA displayed no histopathological changes in the brain. The four remaining animals displayed histopathological lesions in the hippocampus exclusively confined to the CA1 segment (Table 1). Minimal to marked neuronal degeneration and necrosis accompanied by intracellular deposition of a basophilic, birefringent material were observed in the CA1 (Fig. 2a). The birefringent material was demonstrated to contain calcium deposits using von Kossa and Alizarin red stainings (Fig. 2b). The mineralized neurons were also PAS-positive (not shown). Hypertrophic astrocytes were observed in close connection to the degenerating/mineralized neurons within the CA1 area, with weak positive staining for ubiquitin (Fig. 2c) and a moderate increase in GFAP staining (Fig. 2d). A weak positive signal for α-synuclein in astrocytes and a moderate increase in isolectin B4 staining (indicating microglia activation) were also observed in the same region (not shown). Staining for amyloid with Congo red was negative, and the staining pattern/intensity for the TDP-43, tau, and tubulin marker did not differ between vehicle controls and rats treated with BMAA (not shown). In the other studied brain areas, the IHC and special stainings indicated no differences between BMAA-treated rats and vehicle controls. The BMAA-treated rats did not display any histopathological changes in the liver, whereas there was a slight increase in intratubular hyaline droplets in the kidneys compared with the vehicle-treated control animals.

#### Six-month survival time

**Fig. 3:**
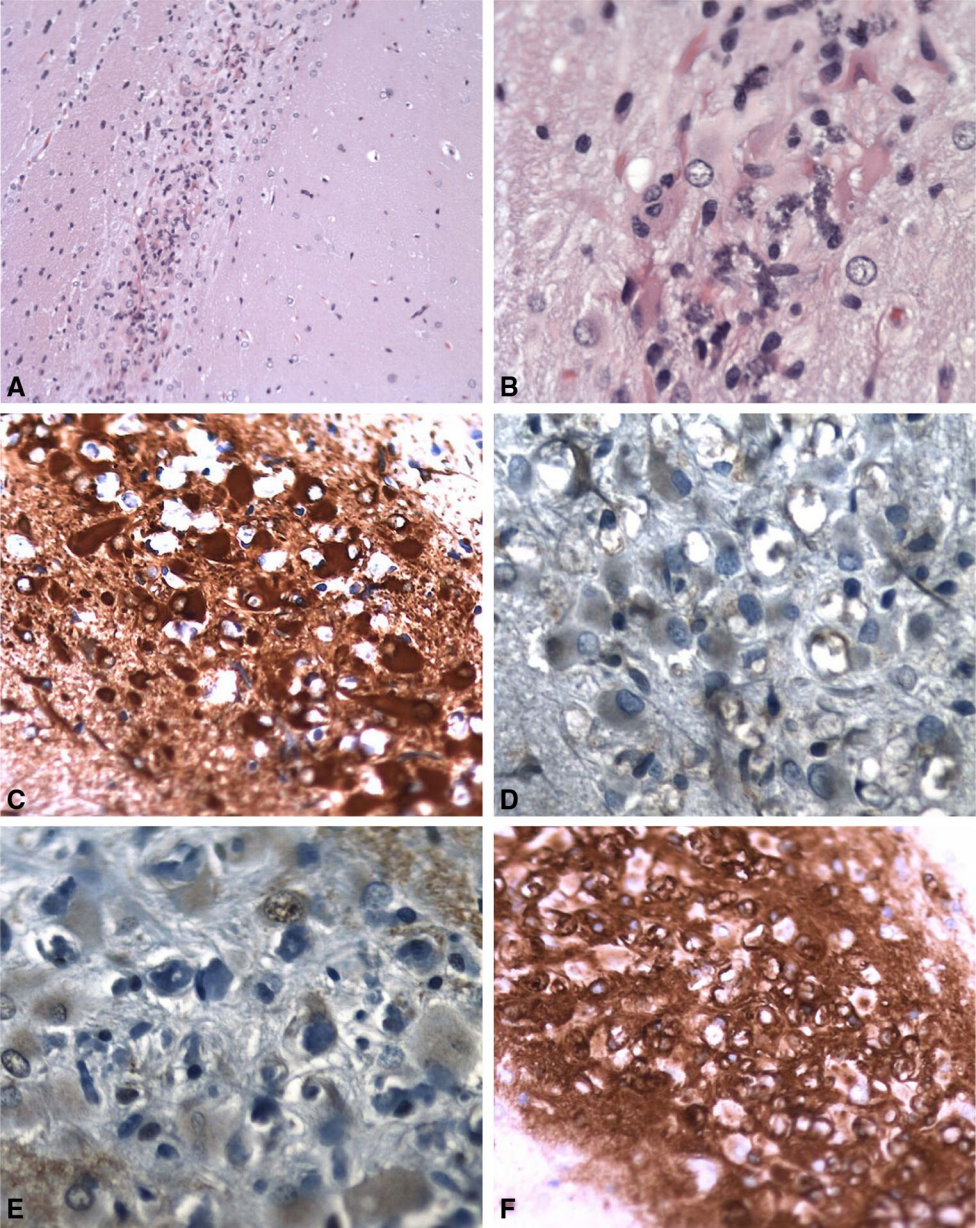
Histopathological changes in the hippocampal CA1 of rats treated neonatally with BMAA on PND 9–10, as examined at the 6-month survival time point. Necrotic neurons are present as basophilic (mineralized) remnants, accompanied by marked astrocytosis (**a**). Some of the astrocytes display intensely eosinophilic cytoplasm, suggestive of Rosenthal fibers (**b**). The astrocytes show positive staining for GFAP (**c**) and ubiquitin (**d**). Magnifications: ×10 (**a**), ×20 (**c**), ×40 (**b**, **d**)

Three out of 8 rats treated with BMAA did not display any histopathological abnormalities in the brain, whereas five rats displayed lesions in the hippocampal CA1 region (Table 1). Mineralization, degeneration, and necrosis of neurons were observed and graded in severity from minimal to marked. In some animals, the CA1 neuronal layer was almost completely obliterated and replaced by hypertrophic astrocytes (Fig. 3a). Some of the astrocytes displayed an intensely eosinophilic cytoplasm reminiscent of the so-called Rosenthal fibers (Fig. 3b). The astrocytes within the CA1 area displayed strong positive staining for GFAP (Fig. 3c) and moderate positive staining for ubiquitin (Fig. 3d) and α-synuclein (Fig. 3e). Increased isolectin B4 staining was also observed in the same region (Fig. 3f). Staining for amyloid with Congo red was negative, and the staining pattern/intensity for the TDP-43, tau, and tubulin marker did not differ between vehicle controls and rats treated with BMAA (not shown). In the other brain areas, the IHC and special stainings indicated no differences between the BMAA-treated rats and vehicle controls. The BMAA-treated rats did not display any histopathological changes in the liver and kidneys compared to the vehicle-treated control animals.

### Ultrastructural examination of neurons and astrocytes in the hippocampal CA1

**Fig. 4:**
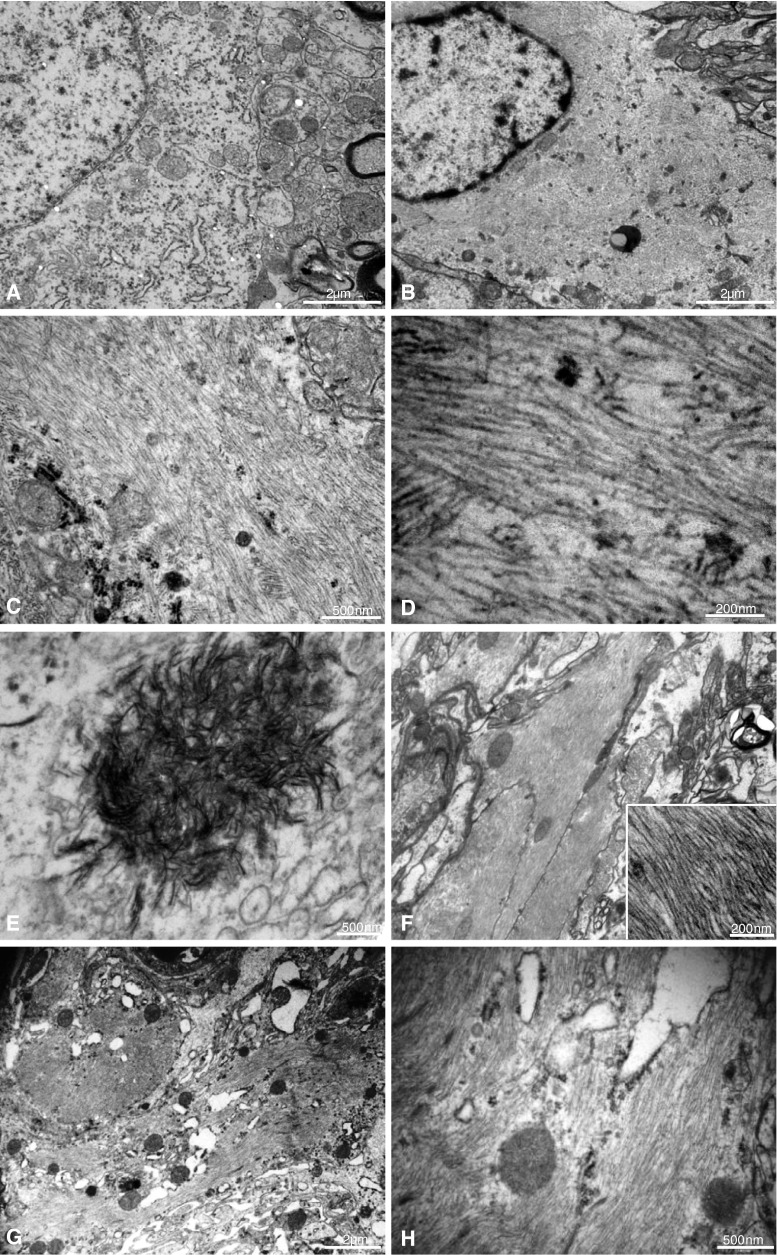
Ultrastructural features of neurons and astrocytes in the hippocampal CA1 of rats treated neonatally with BMAA on PND 9–10, as examined at the 3-month survival time point. A normal control neuron is shown for comparison (**a**). Bundles of parallel-oriented fibrils are present in the cytoplasm of neurons without (**b**–**d**) and with (**e**) intracellular crystals. The appearance of the crystals is consistent with calcium. The fibrils are present also in axons (**f**). In addition, an abundance of fibrils is present in the cytoplasm of astrocytes (**g**–**h**). Magnifications: ×5,800 (**g**) ×7,900 (**a**, **b**, **f**), ×25,000 (**c**, **h**), ×46,000 (**e**), ×92,000 (**d**, **f**; *inset*)

In ultrathin sections of the hippocampal CA1 from representative BMAA-treated rats at the 3- and 6-month survival time points, there were two major and distinct findings: intracellular aggregates of needle-like, electron-dense bodies (crystals), and extensive fibril formation in the perikarya (Fig. 4a–h). Intracytoplasmic bundles of closely packed parallel-oriented fibrils, each with a diameter of approximately 10 nm, were abundant in neurons without (Fig. 4b–d) and with intracellular crystals (Fig. 3e). The ultrastructural features of the crystals were consistent with calcium deposits. The fibrils were present both in the neuronal perikarya (Figure b–d) and in axons (Fig. 4f). Astrocytes also contained an abundance of closely packed fibrils, similar to those observed in the neurons, but crystals were not conclusively identified in astrocytes (Fig. 4g–h). No extracellular crystals or fibrils were detected in the CA1 area.

### Identification of proteins enriched in the histopathologically altered area of the hippocampal CA1 by proteomic analysis

Histopathologically altered areas of hippocampus CA1, containing birefringent material, and ultrastructurally packed with fibrils from brain cryosections of a 6-month BMAA rat treated neonatally were isolated using laser capture microdissection. Adjacent histologically normal CA1 areas on the same brain cryosections were captured as a control. The LC-MS/MS analysis detected 155 proteins in the control area and 203 proteins in the histopathologically altered area. Proteins that displayed higher levels in the control area as well as proteins that were only identified with one peptide were excluded. Finally, 120 proteins were considered to be enriched (at least 1.8-fold) or exclusively detected in the histopathologically altered areas in the hippocampal CA1 (Supplemental Table 2). Several of these proteins are cytoskeletal components, intermediate filaments, and chaperones and are implicated in protein aggregation and fibril formation. Some of these 120 proteins, such as GFAP, connexin 43, phosphoglycerate kinase 1, alpha-enolase, and superoxide dismutase (SOD 1), are also markers of astrogliosis or involved in the energy metabolism and the antioxidant defense system. The most enriched proteins (i.e., plectin, GFAP, vimentin, heat shock protein (Hsp 27), together with some specific proteins that are of special interest for neurodegenerative changes) are shown in Table 2.

**Table 2:** List of proteins enriched in a sample of histologically altered hippocampal CA1 areas of a rat treated neonatally with BMAA on PND 9–10, as examined at the 6-month survival time point

Protein	Mw kDa	Control^a^	Sample^a^	Abundance ratio^b^
Rosenthal fiber-associated proteins				
Plectin	519.7	2	48	24
Glial fibrillary acid protein (GFAP)	49.9	17	133	7.8
GFAP epsilon (fragment)	18.5	1	16	16
Vimentin	53.7	3	68	22.7
Heat shock protein (Hsp) 27	21.7	–	12	Exclusively detected
Other inclusion body-associated proteins				
Alpha-enolase	47.1	2	11	5.5
Apolipoprotein E	35.7	1	7	7
TAR DNA-binding protein 43 (TDP-43)	44.5	–	2	Exclusively detected
Chaperons				
Endoplasmin (GRP-94)	92.7	1	6	6
Clusterin (apolipoprotein J)	51.4	–	3	Exclusively detected
Glucose-regulated protein 78 (GRP-78)	72.3	2	8	4
Autophagy				
GLIPR-2 (GAPR-1)	16.7	–	6	Exclusively detected
Cytoskeletal proteins				
Actin, cytoplasmic 1 (beta-actin)	41.7	34	67	2
Actin, alpha cardiac muscle 1	42	26	47	1.8
Macrophage-capping protein	38.8	–	3	Exclusively detected
Actin-depolymerizing factor (ADF; destrin)	18.5	2	4	2
Antioxidant system				
Glutathione S-transferase mu 1	26	–	6	Exclusively detected
Glutathione S-transferase alpha-3	25.3	–	5	Exclusively detected
Peroxiredoxin-1	22.1	–	4	Exclusively detected
Peroxiredoxin-6	24.8	–	2	Exclusively detected
Protein DJ-1 (PARK7)	20	2	5	2.5
Superoxide dismutase (SOD1)	15.7	–	2	Exclusively detected
Lipid associated proteins				
Fatty acid- binding protein (FABP7)	14.9	–	6	Exclusively detected
Annexin A1	38.8	–	2	Exclusively detected
Annexin A2	38.7	–	4	Exclusively detected
Annexin A3	36.3	–	11	Exclusively detected
Annexin A4	35.8	–	4	Exclusively detected
Annexin, anxa5	33.9	–	3	Exclusively detected
Glia markers and gliosis				
Excitatory amino acid transporter (EAAT) 1	59.6	–	2	Exclusively detected
EAAT 2	60.9	3	6	2
Gap junction alpha-1 protein (connexin 43)	43	1	6	6
Histone H3	15.4	1	6	6
Histone H2B	14	2	17	8.5
Histone H1.4	22	1	9	9

The CA1 sample containing birefringent material was compared with an adjacent CA1 sample not containing birefringent material

A neonatal rat on PND 9–10 was injected sc with BMAA (460 mg/kg) and killed 6 months later. Twenty micrometer cryosections of the brain were used for laser capture microdissection and LC–MS/MS of the hippocampal CA1

^a^The number of different peptides detected for each protein (indicative of protein abundance) in the sample and the control, respectively

^b^The abundance ratio of proteins from the sample versus the control

## Discussion

In this study, we report the intriguing intracellular deposition of bundles of parallel fibrils and calcifications in the hippocampal CA1 of adult rats exposed to the nonprotein amino acid BMAA during the neonatal period on PND 9–10. Neurons and astrocytes contained an abundance of closely packed fibrils, whereas calcium crystals appeared to be confined to neurons only. The bundles of fibrils most likely interfere with cellular and axonal function because of their abundance, and they appear to be resistant to degradation, suggesting that they are permanent changes at this site. The proteomic analysis of the affected site revealed an enrichment of chaperones, cytoskeletal, and intermediate filament proteins that previously have been implicated in protein aggregation and fibril formation. No histopathological damage was observed 2 weeks after BMAA exposure, whereas distinct pathological changes in the hippocampus were present 3 and 6 months after BMAA exposure. Thus, the neurodegeneration, astrogliosis, and microglial activation in the CA1 area were progressive in nature, affecting more animals and displaying increased severity at later survival time points. Still, 3 out of 8 rats treated with BMAA did not display any distinct histopathological abnormalities in the hippocampus 6 months after exposure indicating an interindividual response to BMAA in the outbred Wistar rats with variation in their genome.

Intracellular deposition of fibrillar aggregates characterizes several neurodegenerative diseases, but induction of intracellular fibril formation in vivo by exogenous agents is a rare finding, even though environmental factors are believed to play a major role in these complex disorders (Coppede et al. 2006). To our knowledge, this is the first ultrastructural observation of a nonprotein amino acid that induces extensive intracellular fibril formation in neurons and astrocytes (Bell 2003; Rodgers 2013; Rubenstein 2000). Many proteins exhibit propensities to form fibrils during in vitro conditions, but fibril formation in vivo is often because of misfolded or abnormally processed proteins that have aggregated. Recent studies have indicated that BMAA may be misincorporated into proteins (Dunlop et al. 2013; Karlsson et al. 2009a, 2014). Interestingly, other amino acid analogs that are suggested to be misincorporated into proteins during synthesis may result in accumulation of misfolded proteins in cultured cells in vitro (Dasuri et al. 2011; Dunlop et al. 2008; Rodgers and Shiozawa 2008). The l-tyrosine mimetic l-DOPA has also been reported to be incorporated into proteins of Parkinson’s disease patients treated with l-DOPA (Chan et al. 2012; Rodgers et al. 2006). In addition, Lee et al. (2006) has demonstrated that low levels of mischarged tRNA cause intracellular accumulation of misfolded proteins and neurodegeneration in mice. Misincorporation of BMAA into proteins may therefore be a primary event resulting in fibril formation and neurodegeneration. Although it is not known whether the BMAA-induced fibrils are harmless or pathologic, the progression of the BMAA-induced lesions could indicate that potential misfolded proteins may seed misfolding of other proteins or serve to nucleate additional proteins, forming larger, potentially toxic protein aggregates (Costanzo and Zurzolo 2013; Murphy 2002).

BMAA was identified in cycad seeds and demonstrated to be neurotoxic several decades ago because of its suggested role in the etiology of the progressive neurodegenerative disorder amyotrophic lateral sclerosis/Parkinsonism-dementia complex (ALS/PDC) on the island of Guam (Banack and Cox 2003; Spencer et al. 1987). Several migrants from Guam have developed ALS/PDC decades after they have left the island. This lead Garruto and coworkers to suggest that the etiological process had occurred in utero, during infancy, childhood, or adolescence (Garruto et al. 1980, 1984). Furthermore, it has been reported that exposure to cycads during young adulthood, but not adulthood, is a risk factor for neurodegenerative disorder among the inhabitants of Guam (Borenstein et al. 2007). BMAA has a low neurotoxic potency in adult rodents (Cruz-Aguado et al. 2006; Perry et al. 1989) compared to young animals (Karamyan and Speth 2008; Karlsson et al. 2011). Taken together, the developing brain seems to be more vulnerable to BMAA. The exposure period PND 9–10 used in the present study corresponds to the last trimester of pregnancy and the first few years of age in humans and is characterized by rapid maturation of neuronal systems (Dobbing and Sands 1979). Our recent studies showing that BMAA is transferred via milk to suckling pups are therefore of particular interest for human risk assessment because they raise the possibility that exposure of breast-fed infants to BMAA could occur (Andersson et al. 2013).

ALS/PDC is a severe tangle-forming disorder that affects both neuronal and glial cells (Miklossy et al. 2008). The neuropathology is characterized by neurofibrillary tangles of paired helical filaments (PHFs) composed of abnormally hyperphosphorylated forms of the protein tau, similar to those in Alzheimer’s disease (Miklossy et al. 2008). Excitotoxic amino acids such as glutamate and aspartate are reported to induce PHFs in explant cultures of human neurons (De Boni and McLachlan 1985). Arif et al. (2014) recently found a decrease in protein phosphatase 2A (PP2A) activity associated with abnormal hyperphosphorylation of tau in brain of ALS/PDC patients. Interestingly, BMAA was reported to induce similar changes after icv exposure of neonatal rats by activating mGluR5 receptors (Arif et al. 2014). Although the IHC for tau in the present study failed to demonstrate a distinct increased staining in the histopathologically altered CA1 region, it cannot be excluded that hyperphosphorylation of tau could also play a role in the fibril formation in hippocampal CA1 neurons. However, BMAA-induced PP2A inhibition may also lead to hyperphosphorylation of other proteins that potentially could also result in intracellular fibril formation.

In addition to fibril formation, mineralization was detected in the CA1 neurons as demonstrated by staining with Alizarin red and von Kossa stains. Intraneuronal precipitation of calcium is known to occur following excessive activation of excitatory amino acid receptors in the brain, and it has been linked to Alzheimer’s disease and ALS/PDC (Garruto et al. 1984; Rodriguez et al. 2000). This is a likely mechanism for the observed mineralization as BMAA is a glutamate agonist that increases cytosolic calcium concentrations in brain slices and cultured neuronal cells (Copani et al. 1991; Cucchiaroni et al. 2010; Rao et al. 2006). Moreover, the glutamatergic receptors and their subunits often have transient peak levels during the first neonatal weeks in rats (McDonald and Johnston 1990; Ripellino et al. 1998). Calcifications have been suggested to serve as a protective buffer against free calcium ions (Mäkinen et al. 2007). Histochemical studies on some human neurological diseases involving neuronal calcification (e.g., Fahr’s disease and Sturge-Weber syndrome) indicate storage of polysaccharides and glycoproteins at the calcification sites (Ando et al. 1999). Indeed, increased PAS staining was detected in mineralized neurons in CA1, confirming the accumulation of glycogen and glycoproteins in these neurons of BMAA-exposed neonatal rats.

Laser capture microdissection and subsequent LC-MS/MS were used to characterize enriched proteins in the histopathologically altered CA1 area. A number of chaperones, cytoskeletal, and intermediate filament proteins were found to be enriched in the affected area, and these proteins have been implicated in protein aggregation and fibril formation. However, fibrils are usually resistant to agents employed to dissolve macromolecules, and the present protocol using trypsin, deoxycholate, and dithiothreitol for the proteomic analysis of the histologically abnormal areas may not necessarily have produced complete lysis and disaggregation, which suggests that there may be additional proteins that are enriched. The enrichment of chaperones, cytoskeletal, and intermediate filament proteins may be a downstream/secondary response to the BMAA-induced intracellular formation of fibrils or the proteins they comprise. Different control systems, such as chaperones, ubiquitin–proteasome system (UPS), and lysosomal autophagy, are active in defending against the hazards caused by the accumulation of misfolded proteins (Lamark and Johansen 2012). The neonatal exposure to BMAA appeared to activate these control systems as there was an enrichment of both chaperones and a recently identified negative regulator of autophagy, GLIPR-2, also known as GAPR-1 (Shoji-Kawata et al. 2013) in the affected hippocampal CA1 area. The latter was exclusively detected in this area. Thus, strategies that enhance autophagy via regulation of GLIPR-2 could possibly be used to reduce BMAA-induced toxicity (Shoji-Kawata et al. 2013). Interestingly, some of the BMAA-induced enriched proteins in the histopathologically altered area, such as plectin, GFAP, vimentin, Hsp 27, and ubiquitin, are known to form complex astrocytic inclusions, the so-called Rosenthal fibers, in the neurodegenerative disorder Alexander disease (Hagemann et al. 2009). In addition, some of the astrocytes displayed an intensely eosinophilic cytoplasm, which is also suggestive of Rosenthal fibers. As with other protein aggregation disorders of the brain, it has not yet been fully resolved if the formation of Rosenthal fibers is harmful or protective (Ross and Poirier 2005). Experimental studies have demonstrated that these fibers can originate from either expression of mutant GFAP or over-expression of wild-type GFAP and that elevation in total levels of GFAP may be a critical element in the pathogenesis of Alexander disease (Hagemann et al. 2009; Jany et al. 2013).

Several chaperons, heat shock proteins, including Hsp 27, endoplasmin (GRP-94), clusterin (apolipoprotein J), and GRP-78, which regulate nascent folding and modulate the fate of unstructured or misfolded proteins, were also found to be enriched in the affected CA1 area (Parcellier et al. 2003). Clusterin participates in Aβ, PrP(res), and α-synuclein aggregation in Alzheimer’s disease, prionpathies, and α-synucleinopathies, respectively (Ferrer et al. 2005; Howlett et al. 2013; Sasaki et al. 2002). In addition, other inclusion body-associated proteins, such as the tauopathy-related protein alpha-enolase (Yang et al. 2008), apolipoprotein E, actin, the actin regulatory, macrophage-capping protein, and actin-depolymerizing factor (ADF), also called destrin (Hirano 1994; Minamide et al. 2000), were found to be enriched.

The proteomic analysis of the histopathologically altered hippocampal CA1 also revealed an enrichment of proteins important for the antioxidant defense system, which confirms the ability of BMAA to induce oxidative stress (Liu et al. 2009; Okle et al. 2012). In addition, there was an enrichment of glutathione S-transferase as well as peroxiredoxin, PARK7, and SOD1 in the damaged CA1 area, which indicates an activation of the antioxidant system in the brain of adult rats neonatally treated with BMAA. Furthermore, TDP-43, which is a major constituent of the proteinaceous inclusions that are characteristics of ALS (Lee et al. 2012) and found in ALS/PDC (Maekawa et al. 2009; Miklossy et al. 2008), was exclusively detected in the affected CA1 area. This is in line with our preliminary observation on fibril formation (Brittebo et al. 2012) and recent studies suggesting that BMAA may induce TDP-43 in neonatal rats and SH-SY5Y human neuroblastoma cells (de Munck et al. 2013; Munoz-Saez et al. 2013). However, the presence of TDP-43 in the affected CA1 area could not be confirmed with IHC and the antibody used in the current study.

In our previous study, hippocampal CA1 neurons of BMAA-treated rats displayed increased staining for both ubiquitin and α-synuclein compared with controls (Karlsson et al. 2012). This result could not be reproduced in the present study, and an increased staining for these two markers was only found in astrocytes. The reason for this discrepancy is most likely related to differences in methodology. In the first study, cryosections were used to preserve mineralized neurons. In the present study, we used formalin-fixed, paraffin-embedded tissue sections to obtain better morphological resolution. As a consequence, many mineralized and damaged neurons were lost from the slides, precluding a reliable assessment of ubiquitin and α-synuclein IHC staining in these cells. Judging from the staining intensity in astrocytes in the brain of adult rats treated neonatally with BMAA, the increase in ubiquitin as well as α-synuclein is a late event in the pathogenesis of BMAA-induced neurotoxicity and is not clearly evident until the 6-month survival time point. Accumulation of α-synuclein, involving not only neurons but also astrocytes, has been observed in the brain of Guamanian ALS/PDC patients (Sebeo et al. 2004). In a series of in vitro studies, an interesting link between α-synuclein and calcium has recently been demonstrated. Calcium promoted α-synuclein aggregation both by itself (Nath et al. 2011) and cooperatively with oxidative stress (Goodwin et al. 2013).

Enriched levels of excitatory amino acid transporter (EAAT) 1 and 2, glial fibrillary acidic protein (GFAP), vimentin, and connexin 43, which are markers of glia cells and astrogliosis, were detected by LC-MS/MS in the affected CA1. In addition, the increase in several histones confirms our previous MALDI IMS study showing an increased expression of histones H2 and H3 in CA1 (Karlsson et al. 2012) and corresponds to the marked astrogliosis and activation of microglia at this site observed by IHC. The activated glia and over-expression of their secreted cytokines could start a self-propagating cycle leading to neurotoxicity, which may be one additional mechanism behind the observed progression of the lesions in the BMAA-treated animals. Finally, brain lipid-binding protein (FABP7), which is known to modulate astrocyte function, was enriched in the affected CA1 area (Kipp et al. 2011). Several annexins, also called lipocortins, were also enriched. Annexins are characterized by their ability to bind phospholipids and could suppress phospholipase A_2_. Therefore, it is important to study the effect on lipids and fatty acids in the BMAA-treated animals.

## Conclusions

Considerable evidence supports a multifactorial etiology involving both environmental and genetic factors in neurodegenerative disease. Several genetically engineered mouse models of neurodegenerative disease are available to study protein aggregation processes and behavioral impairments. However, the sporadic formation of fibrils in vivo is less studied. The present study demonstrates that neonatal exposure to the excitotoxic nonprotein amino acid BMAA may offer a novel animal model for the study of fibril formation in the hippocampus in vivo. The critical cellular perturbations preceding fibril formation, such as excitotoxicity, oxidative stress, misincorporation of BMAA into proteins, or other processes, remains to be elucidated. Although several caveats must be noted when comparing the BMAA-induced intracellular formation of fibrils with the pathology in human neurodegenerative disease, the present data suggest that further studies to elucidate the key molecular initiating event of BMAA-induced long-term neurodegenerative effects are warranted.

## Electronic supplementary material

Below is the link to the electronic supplementary material.

Supplementary material 1 (PDF 24 kb)
