# Supplementary material for: Intracellular fibril formation, calcification, and enrichment of chaperones, cytoskeletal, and intermediate filament proteins in the adult hippocampus CA1 following neonatal exposure to the nonprotein amino acid BMAA
Source: Arch Toxicol. 2014 May 6;89(3):423–36. doi: 10.1007/s00204-014-1262-2 (PMC4335130; doi:10.1007/s00204-014-1262-2)
Supplement: Supplementary file 1 — Supplementary material 1 (PDF 24 kb) [file 204_2014_1262_MOESM1_ESM.pdf]

Archives of Toxicology

Intracellular fibril formation, calcification and enrichment of chaperones, cytoskeletal and intermediate filament proteins in the adult hippocampus CA1 following neonatal exposure to the non-protein amino acid BMAA

Oskar Karlsson*, Anna-Lena Berg, Jörg Hanrieder, Gunnel Arnerup, Anna-Karin Lindström, Eva B Brittebo

*****Uppsala University, Department of Pharmaceutical Biosciences, Box 591, SE-751 24 Uppsala, Sweden, E-mail: Oskar.Karlsson@farmbio.uu.se, Fax: +46-18-4714253

Supplementary Material

Table 1 Details of primary and secondary antibodies, and detection systems used in the immunohistochemistry

**Primary Antibody/ dilution**	**Manufacturer/ product number**	**Antigen retrieval**	**Secondary antibody/ dilution**	**Detection system**
Alpha-synuclein 1/500	Abcam/ ab87599	NA	OmniMap Rb (Roche)/ prediluted	Chromo-Map DAB kit (Ventana/ Roche)
GFAP 1/2000	DAKO/ Z0334	Protease 1, 8 min	Goat anti Rabbit biotin immunoglobin (prod no E0432 Dako)/ 1/150	DAB-Map kit (Ventana/ Roche)
Isolectin B4 Biotin conjugated 1/100	Vector/ B-1205	Mw 10 min pH6 citrate buffer	NA	Streptavidin HRP (Dako)
TARDBP (TDP-43) 1/1000	Abcam/ ab42474	Protease 2, 8 min	OmniMap Rb (Roche)/ prediluted	Chromo-Map DAB kit (Ventana/ Roche)
Tau protein 1/2000	Invitrogen/ AHB0042	SCC1	Goat anti Mouse biotin immunoglobin (prod no E0433 Dako)/ 1/250	DAB-Map kit (Ventana/ Roche)
Tubulin	Abcam/ ab6046	SCC2	OmniMap Rb (Roche)/ prediluted	Chromo-Map DAB kit (Ventana/ Roche)
Ubiquitin 1/100	Abcam/ ab7780	SCC1	OmniMap Rb (Roche)/ prediluted	Chromo-Map DAB kit (Ventana/ Roche)

Supplemental Table 2: Proteins enriched in of histologically altered hippocampal CA1 area*

Accession	Proteins	MW [kDa]	Control[1]	Sample[1]	Abundance ratio
Q6S3A5	Plectin 1 [Q6S3A5_RAT]	519,7	2	48	24,0
P31000	Vimentin [VIME_RAT]	53,7	3	68	22,7
A1E252	Glial fibrillary acidic protein (GFAP) epsilon, Fragment [A1E252_RAT]	18,5	1	16	16,0
D3ZEI4	Uncharacterized protein [D3ZEI4_RAT]	46,0	1	10	10,0
P15865	Histone H1.4 [H14_RAT]	22,0	1	9	9,0
Q00715	Histone H2B type 1 [H2B1_RAT]	14,0	2	17	8,5
P47819	Glial fibrillary acidic protein [GFAP_RAT]	49,9	17	133	7,8
P02650	Apolipoprotein E [APOE_RAT]	35,7	1	7	7,0
P37397	Calponin-3 [CNN3_RAT]	36,4	1	6	6,0
Q66HD0	Endoplasmin (GRP-94) [ENPL_RAT]	92,7	1	6	6,0
P08050	Gap junction alpha-1 protein (connexin 43) [CXA1_RAT]	43,0	1	6	6,0
D3ZJ08	Histone H3 [D3ZJ08_RAT]	15,4	1	6	6,0
P04764	Alpha-enolase [ENOA_RAT]	47,1	2	11	5,5
O08651	D-3-phosphoglycerate dehydrogenase [SERA_RAT]	56,5	1	5	5,0
P06761	78 kDa glucose-regulated protein [GRP78_RAT]	72,3	2	8	4,0
D3ZHY7	Microtubule-associated protein (Map4) [D3ZHY7_RAT]	95,0	2	8	4,0
Q6IFZ4	Type II keratin Kb15 [Q6IFZ4_RAT]	57,6	1	4	4,0
P07335	Creatine kinase B-type [KCRB_RAT]	42,7	9	33	3,7
P42123	L-lactate dehydrogenase B chain [LDHB_RAT]	36,6	2	7	3,5
P16617	Phosphoglycerate kinase 1 [PGK1_RAT]	44,5	2	7	3,5
Q6P7S0	Pyruvate kinase [Q6P7S0_RAT]	52,9	6	19	3,2
P35213	14-3-3 protein beta/alpha (Ywhab) [1433B_RAT]	28,0	1	3	3,0
P61983	14-3-3 protein gamma (Ywhag) [1433G_RAT]	28,3	1	3	3,0
P38650	Cytoplasmic dynein 1 heavy chain [DYHC1_RAT]	531,9	1	3	3,0
D3ZC88	Elongation factor 1-alpha [D3ZC88_RAT]	50,1	4	12	3,0
P05197	Elongation factor 2 [EF2_RAT]	95,2	1	3	3,0
P61980	Heterogeneous nuclear ribonucleoprotein K [HNRPK_RAT]	50,9	1	3	3,0
F1LMU0	Myosin-4 [F1LMU0_RAT]	222,7	2	6	3,0
B1WBU9	Phosphorylase [B1WBU9_RAT]	97,3	1	3	3,0
P67779	Prohibitin [PHB_RAT]	29,8	1	3	3,0
B0K023	Rpl7 protein, Fragment [B0K023_RAT]	26,2	1	3	3,0
Q8CFD0	Sideroflexin-5 [SFXN5_RAT]	37,3	1	3	3,0
Q3ZB99	Tight junction protein 2 [Q3ZB99_RAT]	131,3	1	3	3,0
D3ZTF3	Uncharacterized protein [D3ZTF3_RAT]	32,9	1	3	3,0
O88767	Protein DJ-1 (Park7) [PARK7_RAT]	20,0	2	5	2,5
P09117	Fructose-bisphosphate aldolase C [ALDOC_RAT]	39,3	7	17	2,4
P11980	Pyruvate kinase isozymes M1/M2 [KPYM_RAT]	57,8	9	21	2,3
P04797	Glyceraldehyde-3-phosphate dehydrogenase [G3P_RAT]	35,8	12	26	2,2
P30835	6-phosphofructokinase [K6PL_RAT]	85,3	2	4	2,0
D3ZZ99	Alpha-adducin [D3ZZ99_RAT]	73,1	1	2	2,0
F1LPM4	Amine oxidase [flavin-containing] B (Maob) [F1LPM4_RAT]	56,9	1	2	2,0
G3V936	Citrate synthase [G3V936_RAT]	51,8	1	2	2,0
Q7M0E3	Actin-depolymerizing factor (Destrin) [DEST_RAT]	18,5	2	4	2,0
Q62952	Dihydropyrimidinase-related protein 3 [DPYL3_RAT]	61,9	2	4	2,0
Q9JHL4	Drebrin-like protein [DBNL_RAT]	48,6	1	2	2,0
G3V6R2	Excitatory amino acid transporter (EAAT) 2 [G3V6R2_RAT]	60,9	3	6	2,0
B4F772	Heat shock 70 kDa protein [B4F772_RAT]	94,2	1	2	2,0
P10111	Peptidyl-prolyl cis-trans isomerase A [PPIA_RAT]	17,9	1	2	2,0
P35704	Peroxiredoxin-2 [PRDX2_RAT]	21,8	1	2	2,0
F1LRX5	Protein Sptbn1, Fragment [F1LRX5_RAT]	267,7	5	10	2,0
P52873	Pyruvate carboxylase, mitochondrial [PYC_RAT]	129,7	2	4	2,0
Q64548	Reticulon-1 [RTN1_RAT]	83,0	1	2	2,0
Q64428	Trifunctional enzyme subunit alpha, mitochondrial [ECHA_RAT]	82,6	1	2	2,0
D4A786	Uncharacterized protein [D4A786_RAT]	6,4	1	2	2,0
P60711	Actin, cytoplasmic 1 [ACTB_RAT]	41,7	34	67	2,0
P68035	Actin, alpha cardiac muscle 1 [ACTC_RAT]	42,0	26	47	1,8
P62250	40S ribosomal protein S16 [RS16_RAT]	16,4		2	Exclusively detected
P62909	40S ribosomal protein S3 [RS3_RAT]	26,7		3	Exclusively detected
P62755	40S ribosomal protein S6 [RS6_RAT]	28,7		2	Exclusively detected
P62243	40S ribosomal protein S8 [RS8_RAT]	24,2		2	Exclusively detected
D3ZD02	60S ribosomal protein L13 [D3ZD02_RAT]	24,0		3	Exclusively detected
P50878	60S ribosomal protein L4 [RL4_RAT]	47,2		2	Exclusively detected
F2Z3S2	60S ribosomal protein L7a, Fragment [F2Z3S2_RAT]	29,8		3	Exclusively detected
O70371	Annexin, Fragment (Anxa5) [O70371_RAT]	33,9		3	Exclusively detected
P07150	Annexin A1 [ANXA1_RAT]	38,8		2	Exclusively detected
Q07936	Annexin A2 [ANXA2_RAT]	38,7		4	Exclusively detected
P14669	Annexin A3 [ANXA3_RAT]	36,3		11	Exclusively detected
P55260	Annexin A4 [ANXA4_RAT]	35,8		4	Exclusively detected
P15429	Beta-enolase [ENOB_RAT]	47,0		7	Exclusively detected
P09456	cAMP-dependent protein kinase regulatory subunit (Prkar1a) [KAP0_RAT]	43,1		4	Exclusively detected
G3V7K3	Ceruloplasmin [G3V7K3_RAT]	120,6		2	Exclusively detected
D4ACB8	Chaperonin subunit 8 (Cct8) [D4ACB8_RAT]	59,6		3	Exclusively detected
G3V836	Clusterin (apolipoprotein J)[G3V836_RAT]	51,4		3	Exclusively detected
P47875	Cysteine and glycine-rich protein 1 [CSRP1_RAT]	20,6		2	Exclusively detected
P36201	Cysteine-rich protein 2 [CRIP2_RAT]	22,7		2	Exclusively detected
Q68FS4	Cytosol aminopeptidase [AMPL_RAT]	56,1		3	Exclusively detected
Q9JI66	Electrogenic sodium bicarbonate cotransporter 1 [S4A4_RAT]	121,3		2	Exclusively detected
G3V732	Elongation factor 1-delta [G3V732_RAT]	28,7		2	Exclusively detected
G3V846	Excitatory amino acid transporter 1 [G3V846_RAT]	59,6		2	Exclusively detected
P55051	Fatty acid-binding protein, brain [FABP7_RAT]	14,9		6	Exclusively detected
D4A3F5	G protein-coupled receptor (Gprc5b) [D4A3F5_RAT]	44,0		6	Exclusively detected
P04904	Glutathione S-transferase alpha-3 [GSTA3_RAT]	25,3		5	Exclusively detected
B6DYQ1	Glutathione S-transferase mu 1 [B6DYQ1_RAT]	26,0		6	Exclusively detected
D3ZNT4	Heat shock protein beta-1 (Hsp27) [D3ZNT4_RAT]	21,7		12	Exclusively detected
Q4V8K6	Heterogeneous nuclear ribonucleoprotein C (C1/C2) [Q4V8K6_RAT]	32,8		2	Exclusively detected
G3V8L3	Lamin A, isoform CRA_b [G3V8L3_RAT]	74,3		3	Exclusively detected
Q63016	Large neutral amino acids transporter small subunit 1 [LAT1_RAT]	55,9		2	Exclusively detected
Q6P9U7	L-lactate dehydrogenase (Ldha) [Q6P9U7_RAT]	29,8		2	Exclusively detected
Q920P0	L-xylulose reductase [DCXR_RAT]	25,7		2	Exclusively detected
Q6AYC4	Macrophage-capping protein [CAPG_RAT]	38,8		3	Exclusively detected
B2GV33	Maoa protein, Fragment (Maoa) [B2GV33_RAT]	58,8		3	Exclusively detected
P08494	Matrix Gla protein [MGP_RAT]	12,0		2	Exclusively detected
D4ABB2	Mlc1 [D4ABB2_RAT]	44,3		2	Exclusively detected
F1M047	Mitogen-activated protein kinase 3, Fragment (Mapk3) [F1M047_RAT]	42,0		3	Exclusively detected
Q63716	Peroxiredoxin-1 [PRDX1_RAT]	22,1		4	Exclusively detected
O35244	Peroxiredoxin-6 [PRDX6_RAT]	24,8		2	Exclusively detected
D3ZU02	Phosphoglucomutase 3 (Predicted), isoform CRA_d [D3ZU02_RAT]	55,2		2	Exclusively detected
G3V8L9	Polymerase I and transcript release factor (Predicted) [G3V8L9_RAT]	43,9		2	Exclusively detected
D4AC23	Protein Cct7 [D4AC23_RAT]	59,6		2	Exclusively detected
P11598	Protein disulfide-isomerase A3 [PDIA3_RAT]	56,6		2	Exclusively detected
F1M5V2	Protein Glipr2, Fragment (GAPR-1) [F1M5V2_RAT]	16,7		6	Exclusively detected
F1LV13	Protein Hnrpm [F1LV13_RAT]	73,7		4	Exclusively detected
D3ZS68	Protein Pcbp1 [D3ZS68_RAT]	37,5		3	Exclusively detected
D3ZHJ1	Protein Psat1 [D3ZHJ1_RAT]	40,2		3	Exclusively detected
F1LQW3	Protein Sfpq, Fragment [F1LQW3_RAT]	65,7		3	Exclusively detected
D3ZGP5	Protein Tardbp (TDP-43)[D3ZGP5_RAT]	44,5		2	Exclusively detected
P50399	Rab GDP dissociation inhibitor beta [GDIB_RAT]	50,5		4	Exclusively detected
Q5PQK5	Radixin [Q5PQK5_RAT]	68,5		2	Exclusively detected
Q5XI73	Rho GDP-dissociation inhibitor 1 [GDIR1_RAT]	23,4		2	Exclusively detected
B0BN81	Ribosomal protein S5, isoform CRA_b [B0BN81_RAT]	22,9		2	Exclusively detected
P29457	Serpin H1 [SERPH_RAT]	46,5		2	Exclusively detected
Q63555	SP120 (Hnrnpu) [Q63555_RAT]	87,7		3	Exclusively detected
Q6LDS4	Superoxide dismutase [Cu-Zn] (Sod1) [Q6LDS4_RAT]	15,7		2	Exclusively detected
Q7TPB1	T-complex protein 1 subunit delta (Cct4) [TCPD_RAT]	58,1		2	Exclusively detected
Q68FQ0	T-complex protein 1 subunit epsilon (Cct5) [TCPE_RAT]	59,5		2	Exclusively detected
Q2LAP6	Testin [TES_RAT]	47,6		2	Exclusively detected
Q498D4	Tln1 protein [Q498D4_RAT]	161,9		2	Exclusively detected
P37805	Transgelin-3 [TAGL3_RAT]	22,5		2	Exclusively detected
P50137	Transketolase [TKT_RAT]	67,6		2	Exclusively detected
F1LP60	Uncharacterized protein, Fragment [F1LP60_RAT]	67,6		2	Exclusively detected

* A neonatal rat on PND 9-10 was injected sc with BMAA (460 mg/kg) and sacrificed 6 months later. Cryo-sections were used for laser capture microdissection and LC-MS/MS of the hippocampal CA1. The CA1 sample containing birefringent material was compared with an adjacent normal tissue. [1]Number of different peptides detected for each protein (indicative of protein abundance).
